# Deep learning for automated sleep staging using instantaneous heart rate

**DOI:** 10.1038/s41746-020-0291-x

**Published:** 2020-08-20

**Authors:** Niranjan Sridhar, Ali Shoeb, Philip Stephens, Alaa Kharbouch, David Ben Shimol, Joshua Burkart, Atiyeh Ghoreyshi, Lance Myers

**Affiliations:** Verily Life Sciences, Mountain View, CA USA

**Keywords:** Machine learning, Biomarkers

## Abstract

Clinical sleep evaluations currently require multimodal data collection and manual review by human experts, making them expensive and unsuitable for longer term studies. Sleep staging using cardiac rhythm is an active area of research because it can be measured much more easily using a wide variety of both medical and consumer-grade devices. In this study, we applied deep learning methods to create an algorithm for automated sleep stage scoring using the instantaneous heart rate (IHR) time series extracted from the electrocardiogram (ECG). We trained and validated an algorithm on over 10,000 nights of data from the Sleep Heart Health Study (SHHS) and Multi-Ethnic Study of Atherosclerosis (MESA). The algorithm has an overall performance of 0.77 accuracy and 0.66 kappa against the reference stages on a held-out portion of the SHHS dataset for classifying every 30 s of sleep into four classes: wake, light sleep, deep sleep, and rapid eye movement (REM). Moreover, we demonstrate that the algorithm generalizes well to an independent dataset of 993 subjects labeled by American Academy of Sleep Medicine (AASM) licensed clinical staff at Massachusetts General Hospital that was not used for training or validation. Finally, we demonstrate that the stages predicted by our algorithm can reproduce previous clinical studies correlating sleep stages with comorbidities such as sleep apnea and hypertension as well as demographics such as age and gender.

## Introduction

About 50 to 70 million Americans suffer from sleep or wakefulness disorders. As symptoms appear during sleep, they are not easily apparent to patients and most sleep disorders remain undiagnosed^1^. Moreover, sleep deficiency and anomalies in sleep architecture are linked to many chronic health problems, including sleep apnea, diabetes, stroke, brain injury, Parkinson’s disease, depression, and Alzheimer’s disease^2–8^. Therefore measuring sleep behavior can diagnose sleep disorders and also lead to early detection of other health conditions.

Currently, clinical sleep diagnosis requires polysomnography (PSG) study to measure overnight electroencephalogram (EEG), electrooculogram (EOG), electrocardiogram (ECG), airflow, and other signals. Once the data are collected, scoring requires an expert to spend up to 2 hours to analyze and manually annotate each night. Therefore PSG studies are expensive and often only used after significant progression of a patient’s symptoms^1^. These studies may have other disadvantages as well. Laboratory PSG studies can cause significant disruption to the patient’s sleep and fail to capture a patient’s normal sleep patterns^9^. Manual scoring also has considerable interscorer and intrascorer variability, making its reliability and reproducibility questionable^10^. Multiple machine learning algorithms have been devised to automate sleep scoring and recent algorithms have reached near human level scoring using PSG data^11^. While these can make clinical sleep studies more cost efficient and consistent, the absence of an easily accessible and reliable screening mechanism still leaves a large diagnosis gap between doctors and patients. This gap has to be bridged with a technology that can help a patient detect sleep disorder symptoms with minimal disruption yet high enough accuracy to recommend further treatment or consultation with a physician.

Due to the cost and disruptive nature of clinical sleep studies, today they are singular events performed for one night involving subjects already known to be at a high risk of sleep disorders. Such studies can diagnose chronic conditions such as apnea or periodic limb movement which occur many times every night. Even for these conditions, disease progression cannot be easily monitored as it requires repeated studies. Many important symptoms of sleep disorders such as insomnia, sleep fragmentation, rapid eye movement (REM) instability and inadequate deep sleep only have one or few measurements per night which may be insufficient to make conclusive diagnosis about individuals^2^. As a result, despite a growing body of evidence linking sleep stages with a large number of comorbidities^2–8^, most clinical analysis of sleep stages are cross-sectional studies on populations rather than on individual patients^2,12^. This limits the clinical utility of sleep stages. Therefore, novel technology is required to enable low-cost and accurate longitudinal sleep monitoring to determine if patterns in sleep architecture can be used for reliable clinical diagnosis on individuals as well as track disease progression over time.

Cardiac rhythm or heart rate variability is well-known to be modulated by sleep stages^13–15^. If clinically useful sleep scoring can be performed using only cardiac rhythms, then the myriad of existing medical and consumer-grade devices that can measure signals such as ECG or photoplethysmogram (PPG) can fill the diagnosis gap by enabling low-cost sleep evaluations without requiring full PSGs. In this work, we develop a deep learning algorithm which uses the instantaneous heart rate (IHR) time series extracted from ECG as the sole input to predict the full sleep architecture or hypnogram of the subject. A hypnogram can be used to infer many of the key metrics required to diagnose a wide variety of sleep disorders.

## Results

**Fig. 1:**
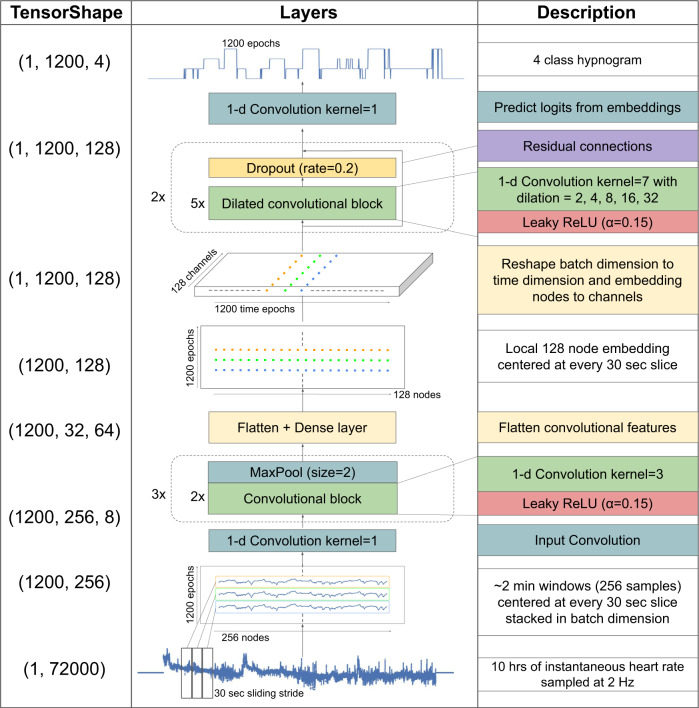
Neural network architecture for sleep stage classification. Instantaneous heart rate, resampled to 2 Hz and padded to 72000 samples (10 h) is used as input. 1200 overlapping patches containing 256 samples each are created, one for every 30 s epoch of the input. Convolutional layers are used to extract local features from each patch to an 128-node embedding layer. The local features are then concatenated to form a vector time-series of length 1200 and depth 128. Next, dilated convolutional layers are used to extract long range temporal features across the length of the vector. Finally, an output convolutional layer is used to output 1200 four-class probability distributions.

**Fig. 2:**
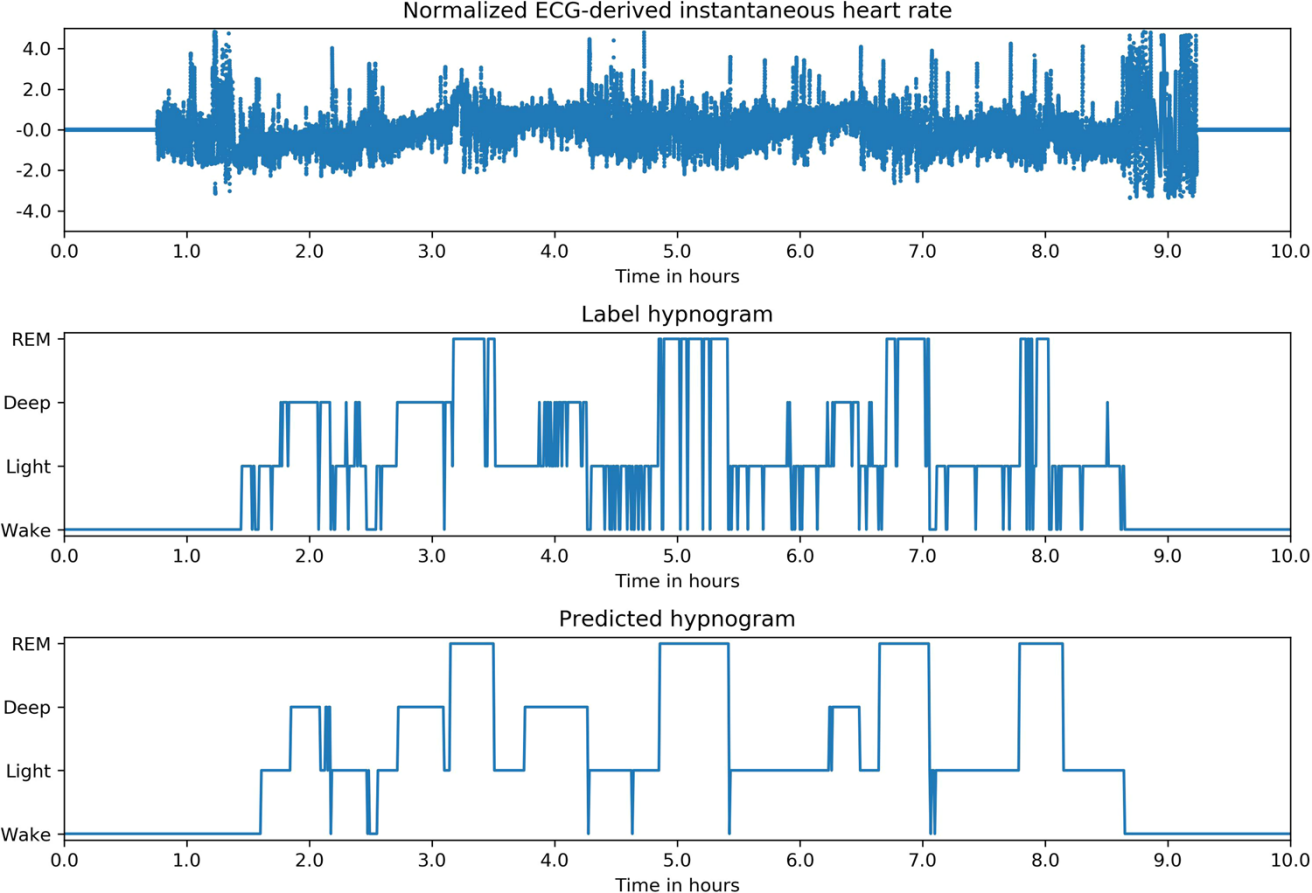
Example of Inputs, labels and predictions. Hypnogram of a healthy subject shows typical 60–90 min sleep cycles with more deep sleep in the first half of the night and more REM in the second half. The predicted stages track the reference very well and are also “smooth” compared with the reference, i.e they have fewer rapid stage bouts that only last 1 or 2 epochs.

We trained a fully convolutional neural network (CNN) which used dilated convolutional blocks to learn both local and long range features of the input (Fig. 1). The network takes as input the IHR extracted from ECG R-wave timing and produces a four-class probability distribution for every non-overlapping 30 s epoch of the input corresponding to the probabilities of the epoch being in one of four classes—wake, light sleep, deep sleep or REM (Fig. 2). The largest probability is chosen as the network’s class prediction and used to form the hypnogram.

We used data from two large public datasets for training, validation and testing of the algorithm, the Sleep Heart Health Study (SHHS)^16^ and Multi-Ethnic Study of Atherosclerosis Study (MESA)^17^. Another independent dataset of 993 nights (993 subjects) from the Physionet Computing in Cardiology (CinC) dataset^18^ was used exclusively as a test set.

### Model performance

**Fig. 3:**
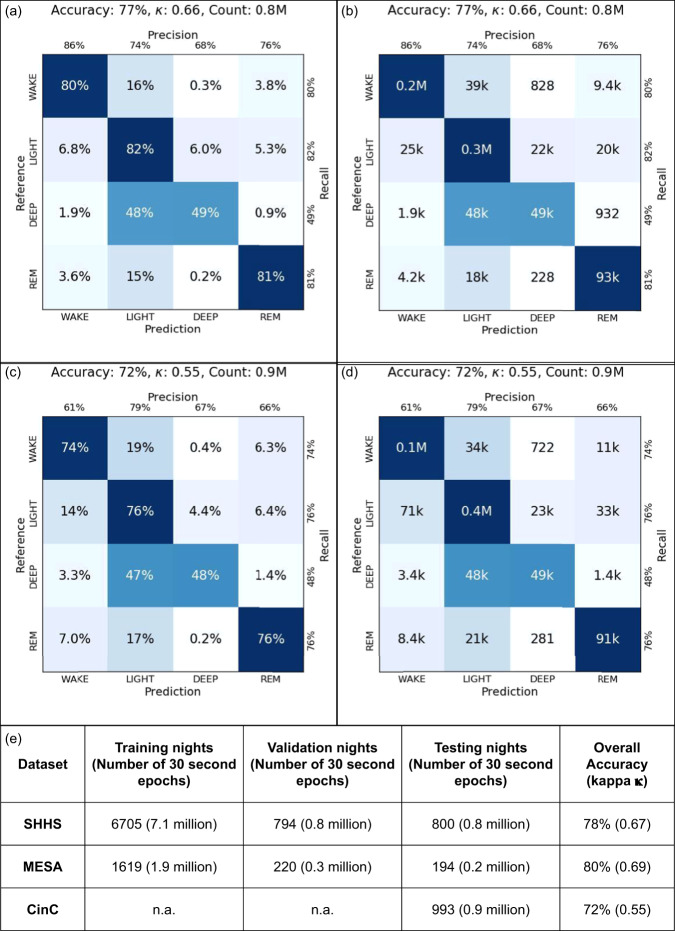
Overall model performance. Confusion matrix for the SHHS dataset (**a**) normalized and (**b**) full counts. Confusion matrix for the CinC dataset (**c**) normalized and (**d**) full counts. (**e**) Dataset sizes and overall accuracy.

On the held out test set of SHHS dataset of 800 nights (561 subjects), the overall 4-class accuracy was 77% and Cohen’s kappa was 0.66. On the Physionet CinC dataset of 993 nights (993 subjects), the overall 4 class accuracy was 72% and Cohen’s kappa was 0.55. The dataset sizes and the model’s overall accuracies and confusion matrices on SHHS and CinC are tabulated in Fig. 3.

**Fig. 4:**
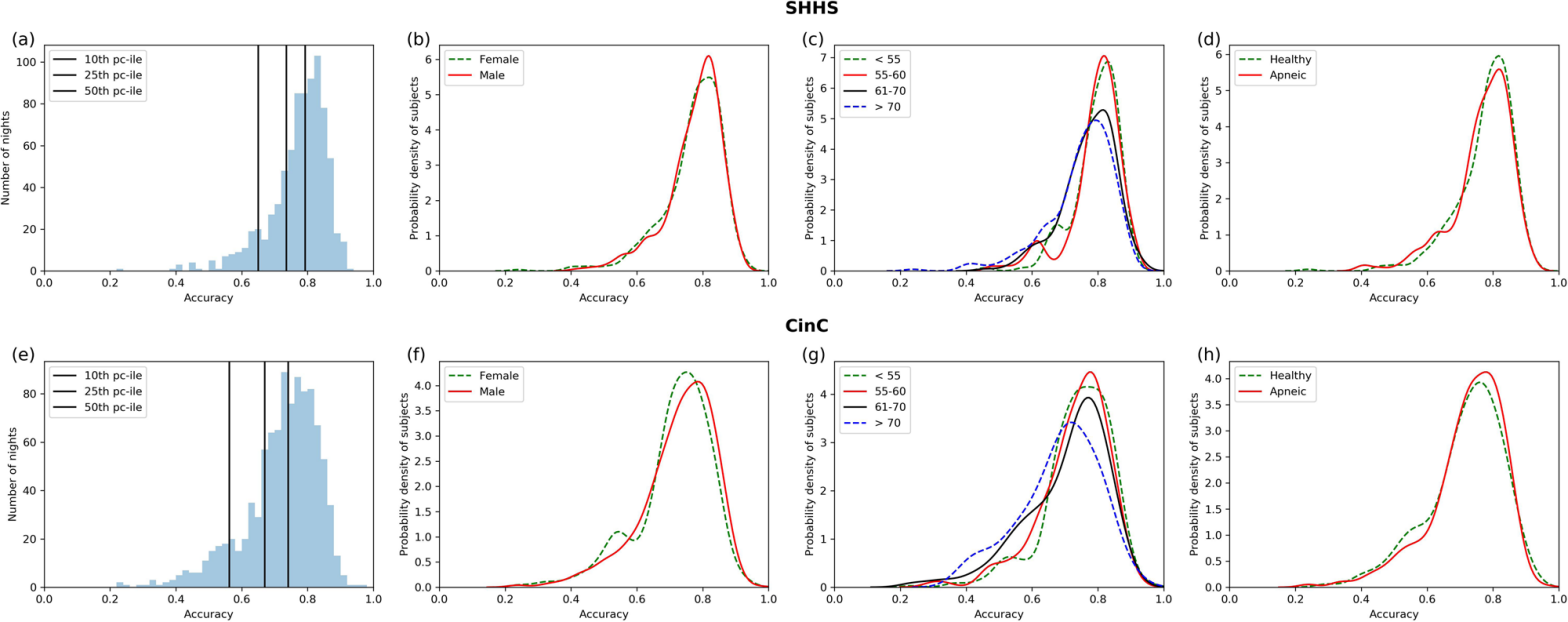
Night by night accuracy distributions on SHHS and CinC datasets. **a**, **e** Histogram of all nights. The black lines mark the 10th, 25th, and the 50 percentile of nights. **b**, **f** Probability density distribution of nightly accuracy split by gender. **c**, **g** Probability density distribution of nightly accuracy split by age quartiles. **d**, **h** Probability density distributions of nightly accuracies split by presence of apnea.

On the held out SHHS dataset, the mean per night accuracy was 77.3% (+/−8.8%) and kappa was 0.65 +/−0.14. On the Physionet CinC dataset, the mean per night accuracy was 72.2% (+/−11.2%) and kappa was 0.53 (+/−0.17). The night by night accuracy histograms are shown in Fig. 4a, b.

The per-class performance (recall, precision) of the algorithm was wake-(0.80, 0.86), light-(0.82, 0.74), deep-(0.49, 0.68), REM-(0.81, 0.76) on SHHS and wake-(0.74, 0.61), light-(0.76, 0.79), deep-(0.48, 0.67), REM-(0.76, 0.66) on CinC.

For comparison, the previously published best 4 class accuracy (kappa) on these datasets was 66% (0.47) on SHHS and 65% (0.31) on CinC using ECG-derived signals^19^.

The best published per-class performance (recall, precision) on these datasets was wake-(0.75, 0.73), light-(0.62, 0.84), deep-(0.59, 0.13), REM-(0.61, 0.39) on SHHS^19^.

Figure 4a, e demonstrate that the model’s per night accuracy is high both on the dataset it was trained on and a new independent dataset. For both these datasets, we then further separate the subjects by gender, age and apnea status. Figure 4b, f show that for both SHHS and CinC test datasets, the model’s performance did not differ between males and females. Figure 4c, g show that the model’s performance decreases on older subjects. Finally, Fig. 4d, h show that the model’s performance does not differ between healthy subjects versus subjects with AHI > 5. Thus the model can be seen to generalize well across different subject populations.

### Clinical outcomes correlation analysis

To demonstrate the clinical efficacy of our model, we show that the stages predicted by our model can reproduce the findings of previous clinical studies^2,12^ which use the expert scored PSG sleep stages. In this section, we summarize the results of our analysis as performed on the held-out test cohort of SHHS dataset. Detailed description of the statistical test and multivariate regression can be found in the “Methods” section.

**Fig. 5:**
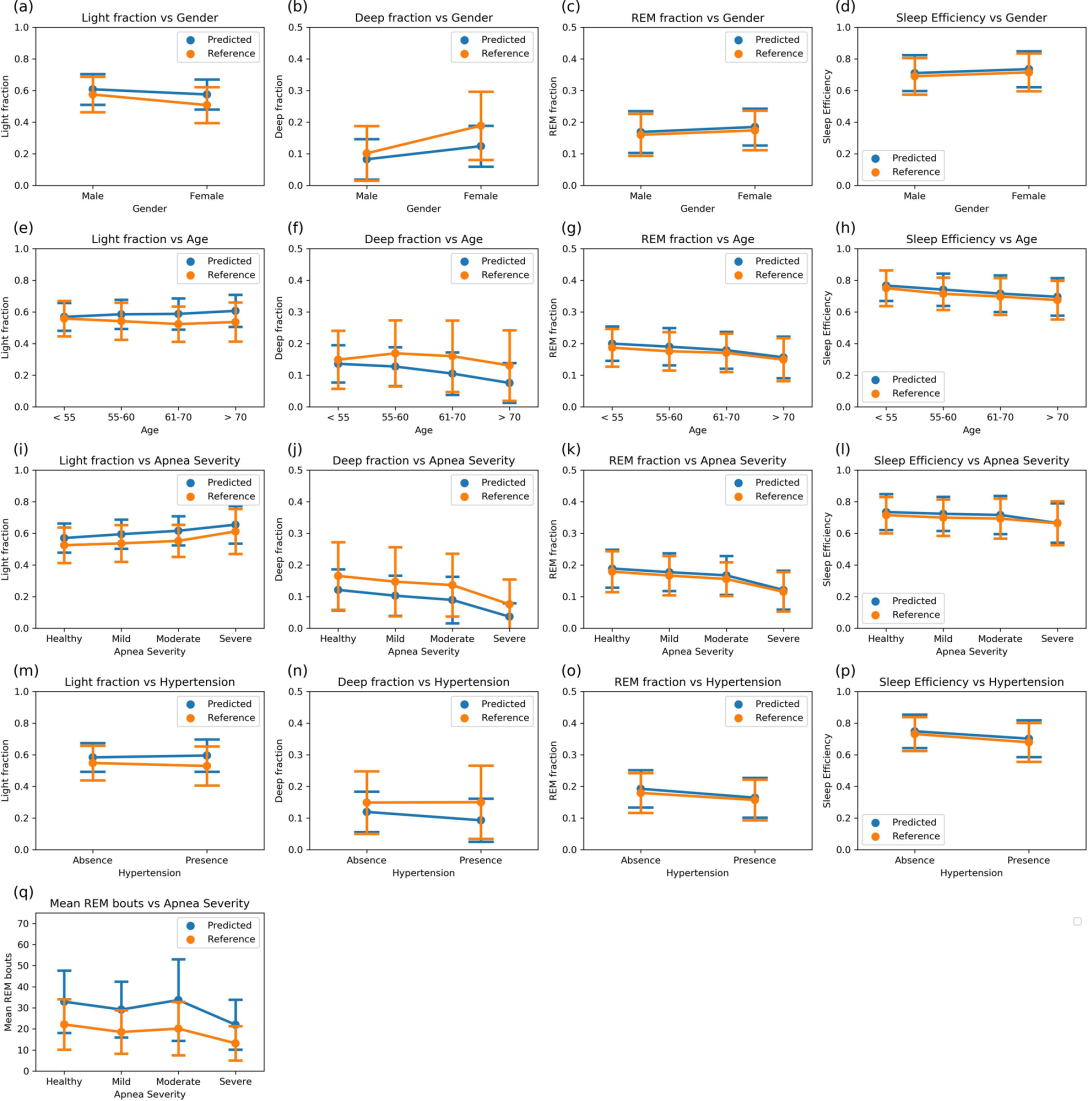
Distributions of sleep metrics derived from PSG reference and model predicted stages. The test population is split into groups by the clinical measures marked in the *x*-axis. The *y*-axis shows the values of sleep metrics—points represent the mean value of the metrics and the bars represent the standard deviations. The distributions calculated using the PSG reference stages and the algorithmically predicted stages show excellent concordance for fraction of REM during sleep and sleep efficiency. Deep sleep is underestimated by our model in favor of light sleep, yet the qualitative effect of clinical states on light and deep fractions is consistent between reference and predicted stages. **a**–**d** stage fractions and sleep efficiency with gender. *N* = 360, 440. **e**–**h** stage fractions and sleep efficiency with age. *N* = 164, 168, 198, 270. **i**–**l** stage fractions and sleep efficiency with apnea severity. *N* = 364, 314, 65, 57. **m**–**p** stage fractions and sleep efficiency with presence of hypertension. *N* = 436, 364. **q** mean REM bout lengths with apnea severity. The mean REM bout durations calculated from algorithmically predicted stages are higher than the PSG reference stages because the predicted stages are “smoother”, i.e., contain fewer stage bouts that last only 1 or 2 epochs interrupting larger stage bouts. Despite this effect, the increase in REM instability seen in apneic subjects is evident. *N* = 364, 314, 65, 57.

Gender—Redline et al.^12^ investigated the variations in expert-labeled PSG sleep stages and their correlation with independent clinical and demographic measures. In their report, men had significantly higher percentage light sleep (N1/N2), had lower percentage deep sleep (N3/N4), percentage REM sleep, and sleep efficiency than women. In our test cohort, there are 360 nights of male subjects and 440 nights of female subjects. We are able to reproduce all the correlations on the test cohort using the sleep stages produced by our algorithm (Fig. 5a–d). We note that the magnitude of the effect on deep sleep is weaker with predicted stages than with reference stages as our algorithm underestimates deep sleep in women.

Age—To study age correlation, we use the same four age groups defined by Redline et al.^12^ which yield 164 nights of subjects aged <55-years old, 168 nights of subjects aged 55–60 years old, 198 nights of subjects aged 61–70 years old, and 270 nights of subjects above the age of 70. Redline et al.^12^ report that percentage light sleep was increased in older individuals along with a corresponding decrease in deep and REM sleep percentage. Sleep efficiency also decreased significantly with increasing age. Once again, we are able to reproduce the expected effects from our predicted stages (Fig. 5e–h). We also see that even though the absolute values of deep sleep are underestimated by our algorithm, the relative effect on predicted deep sleep across age quartiles is significant and comparable to the effect on reference sleep stages.

Sleep Apnea—We divide the subjects into four classes based on apnea severity as measured by the clinical AHI scores—364 nights with AHI score between 0 and 5 (Healthy), 314 nights between 5 and 15 (Mild), 65 nights between 15 and 30 (Moderate) and 57 nights with AHI score greater than 30 (Severe). Bianchi et al.^2^ observe a significant trend of decreasing deep and REM sleep percentage with increasing apnea severity. We also report these effects in both the expert labeled and algorithm predicted sleep stages (Fig. 5i–l). The effects persisted even after adjusting for age and gender.

Bianchi et al.^2^ further identify a novel signature of apnea in sleep stages using stage bout durations. They show that increasing apnea severity in three cohorts, the healthy, mild, and severe apnea cohorts, increased stage transition rate between REM and NREM stages which can be measured as a decrease in mean durations of REM sleep bouts. We find the same effect with our predicted stages (Fig. 5q). We also find that the moderate apnea cohort does not follow the same pattern with the reference PSG stages and our predicted stages correctly identify this behavior. As anticipated by Bianchi et al.^2^, the effects persisted even after adjusting for age and gender.

Hypertension—Redline et al.^12^ reported that subjects with hypertension have lower sleep efficiency than subjects without hypertension. They also report that after adjusting for age, sex and sleep disordered breathing, they find no significant effects of hypertension on light, deep and REM sleep percentage. In our analysis, we used 436 nights from healthy subjects and 364 nights from subjects with a history of taking hypertension medication. We find significant effects on all sleep stage percentages and sleep efficiency using unadjusted algorithm outputs (Fig. 5m–p). The effect on light and deep sleep disappears when adjusted for age, sex and apnea. The effects on REM percentage and sleep efficiency persisted after adjustment.

## Discussion

Patterns in heart rate have long been known to be strongly correlated with sleep cycles^13–15^. The most predictive cardiac features are complex features such as spectral band power ratios and variability measures, which unlike EEG-based features cannot be determined solely by visual inspection, necessitating the use of statistical models. The accuracy of automated cardiac rhythm based sleep staging has been increasing with advances in mathematical and computational techniques as well as cardiology. An important development was made in the field when it was found that the heart rate variability patterns seen during REM often began several minutes prior during NREM and continued well after the REM stage ended^15^. Therefore, temporal context is required to accurately detect sleep stages from cardiac rhythm. The current state-of-the-art results extract dozens, if not hundreds, of expert-informed engineered features from physiological signals such as ECG/PPG and then use these as inputs to complex machine learning models to predict sleep stages^19–22^. The models either use features of neighboring time epochs explicitly to predict the stage for each time epoch^19^ or use implicitly temporal models such as recurrent neural networks (RNNs)^20^. Such models have two important drawbacks that we address with our novel architecture:

1. While hundreds of cardiac features have been developed, it is not clear how to find new features, especially since the strength of the correlation is between cardiac features and PSG sleep stages it is not known a priori. In this work, we do not engineer any features, instead we use convolutional layers to let the network learn local cardiac features. Showing that these networks can exceed the performance of known features can spur research into interpretation of these learned features and their correlation with physiological states.
2. RNN models have a recurrent structure which allows its parameters to be dependent on adjacent parameters, making them very good for representing temporal or otherwise ordered structure in data. However, RNNs, even the so-called long short-term memory or LSTM RNNs, have a fairly short but high fidelity “memory”. They are great for language models where each word may contain a lot of information, but there are not many words in a sentence. In machine learning terms, the number of tunable parameters of an LSTM model scales as *N x L*, where N is the number of layers and L is the length of the timeseries. Our hypothesis is that cardiac rhythm time-series are not as densely packed with information at every 30-s intervals, but they do contain long range temporal context, e.g., 90-min-long sleep cycles. Thus, we estimate that LSTM models are likely to overemphasize short range noise and fail to capture many of the long range correlations. Dilated CNNs address both of these deficiencies.The number of parameters in dilated CNNs scales as N, i.e independent of L^23,24^. Our dilated layers have a field of view ranging from 6 to 90 min with all layers using the same number of parameters. Thus dilated layers are great for capturing long range correlations with far fewer parameters than RNNs.

Therefore our choice of network architecture (local CNN + dilated CNN) is strongly motivated by the characteristics of the problem.

Though the use of additional signals such as airflow, thoracic excursions or more ECG features could potentially have improved accuracy further, we intentionally use only IHR for its practical viability. Specifically, the IHR can be derived from a variety of wearable or passive monitoring sensors such as ECG, wrist-worn or camera-based pulse PPG, bed sensor ballistocardiography (BCG) and potentially others. Many of these sensors are cheaper to produce and far easier to administer than full PSG and some are already used in consumer devices. Therefore if IHR-based sleep staging can be reliable, accurate and can generalize well to new populations, it can unlock access to sleep disorder diagnosis for all.

In this work, we show that our model cannot only achieve high accuracy, but also generalize across multiple datasets, including datasets that were never exposed to the model during training and validation process. We also demonstrate the model’s performance is not biased by gender or presence of apnea in the subjects, and decreases only slightly with increasing age. The datasets we use to demonstrate our results are among the largest used in any previous sleep study, totaling nearly 1987 nights (1748 unique subjects) used for testing.

We also further investigate the potential clinical efficacy of our work by comparing the sleep measures derived from algorithmically determined sleep stages with those derived from the expert-annotated PSG reference stages. We can see that for most of the sleep metrics, our predicted measures closely match the PSG based measures. The algorithmically predicted sleep measures are also able to reproduce all but one clinical effect shown by the reference measures, including null effects.

We hope that these results will help to build more trust in automated heart rate based sleep staging and encourage further research into its clinical application in screening and diagnosis of sleep disorders. Low cost, high efficacy devices which can be used in longitudinal studies on large populations can lead to breakthroughs in clinical applications of sleep staging for early diagnosis of chronic conditions, novel care and treatment endpoints, and improved outcomes for patients.

As mentioned above, our algorithm uses only IHR as input because it can be extracted from a wide variety of medical and consumer devices. Here, we limit our work to heart rate extracted using Pan-Tompkins algorithm from ECG. We note that using alternative sensor input signals (such as PPG) or alternative algorithms for IHR extraction may result in slightly different IHR characteristics. Further analysis is required to quantify the comparative performance of our model on heart rate derived using other signals and algorithms.

We also expect that adding more signals, such as motion and breathing, can increase the accuracy. These signals are usually highly characteristic of the measuring device and its placement on the body. Therefore we hope that our algorithm will serve as a basis for future research involving medical devices that are able to achieve PSG-level sleep staging using cardiac rhythms and other device-specific signals.

Another limitation of our work is that it cannot be used for real-time sleep stage detection, as the model requires a full night of data as an input, prior to quantification.

As noted, our model performs slightly worse on older subjects. While the median accuracy of the oldest cohort was only marginally lower, the fraction of nights with accuracy less than 0.6 is significantly higher in this cohort, especially in the CinC dataset. This could be caused by a variety of reasons—presence of multiple disease confounders, poorer compliance/data quality, lower reference inter-rater reliability—all of which are likely to affect older subjects more. Further research is required to compare the factors affecting sleep studies on different age groups. We hope that our work leads to technologies that make such sleep studies easier and cheaper to administer.

Our model shows good performance on wake, light and REM sleep, but underestimates deep sleep compared with reference stages. One possible reason could be that the inter-rater reliability of expert raters is lowest on deep sleep and is known to vary significantly with age and gender^10,25^. Another possible reason is that the expert scoring for the SHHS dataset was done using the Rechtschaffen and Kales (R&K) scoring guidelines while the MESA and CinC datasets were scored using AASM guidelines^16–18^. The two scoring conventions agree on most epochs but do have some disagreements, especially on light and deep sleep^26^. We chose to use MESA for training and CinC for testing because AASM scoring is the current standard used by sleep physicians. However, SHHS is the largest available PSG dataset with clinical measures and our model gains significant generalizability from training on SHHS and MESA combined. Our strong results despite our model having to generalize across both scoring guidelines demonstrates the capacity of our novel architecture and training strategy. Future efforts can improve upon our results by using new PSG datasets exclusively annotated using AASM guidelines.

Our clinical analysis dataset was limited to the randomly predetermined test split without matched controls. Therefore, we do not attempt novel discoveries with this analysis, we simply demonstrate that our algorithm can reproduce previous investigations that were only accessible using full PSG studies. Furthermore, while we show that we can reproduce the effects of certain clinical conditions on sleep, there are many clinical conditions which are not present in our selected datasets. Further studies will be required to test whether our algorithm can make robust sleep stage predictions in the presence of those conditions.

Finally, an important limitation of our algorithm’s clinical application arises from the lack of explainability of deep neural networks. Traditional machine learning models use engineered features informed by domain expertise as input to simple models making it possible to explain and understand these models’ behavior. Even so, we note that the recent advances in automated sleep scoring have been made using complex models such as Random Forest or LSTM which despite using engineered features are also hard to explain. In this work, we do not explicitly use any engineered features. The algorithm “learned” the features present in raw sequences of heart rate implicitly; therefore it is possible that the algorithm has learnt features previously unknown to or ignored by humans. It would be beneficial to be able to understand these features, however a full understanding of the precise features learnt by deep neural nets is still beyond reach and an active area of research. Though we have not conducted an exhaustive analysis of every aspect of our neural network architecture, several insights such as convolutions to improve training speed over recurrent designs and dilated convolutions to increase the receptive field are consistent with those in prior published work^24^. We hope that our empirical validation puts our work on strong foundation and encourages further research into interpretation and understanding of deep neural networks.

## Methods

### Datasets

Both SHHS and MESA have been archived by the National Sleep Research Resource^27,28^ with appropriate de-identification. Permissions and access for these datasets were obtained via the online portal: www.sleepdata.org. 8299 nights from SHHS and 2033 nights from MESA datasets were processed and used to build and validate the model. The two datasets were mixed and the subjects were randomly separated into training, validation and test sets in ~80:10:10 ratio. The training and validation sets were used to develop the model, while the test set was never exposed to the algorithm during development. Care was taken to ensure that no subjects were shared between the test and training/validation datasets.

The Physionet CinC is archived and available at www.physionet.org, released as part of the Computing in Cardiology Challenge 2018^18^.

### Expert scoring labels

All the datasets used for training, validation and testing were part of full PSG studies. The signals recorded included multi-channel EEG, EOG, electromyogram (EMG), ECG, thoracic and abdomen excursions, airflow and finger pulse oximetry. Each night was then scored for sleep stages and breathing disorders by a sleep expert using full PSG data. Every 30 s is assigned one of 5 classes—wake, N1, N2, N3, and REM. In this work, we group N1 and N2 classes into one class that we call light sleep. We also denote N3 as deep sleep. In SHHS, we have an additional stage N4, which we combine with N3.

For training and testing, we consider the associated labels for each night as the truth reference for the corresponding datasets. Since the datasets were scored by different groups of experts, we must account for the inter-rater accuracy of these labels when judging the performance of the model. We defer to the prior work done to quantify the inter-rater reliability of licensed experts^10,25,26^. Here we simply note two observations. First, many different studies have independently found that human expert inter-rater reliability on the 4-class sleep staging is around 88%^10,25^. Second the above reliability is achieved using full PSG data (i.e., EEG, EOG, EMG, airflow, and others). Therefore our heart rate based model accuracy is not directly comparable to this scoring accuracy. Therefore we benchmark our model against previous efforts to score sleep stages without EEG/EOG data.

### Input features

The heart rate in this effort was extracted from ECG. First we normalize the ECG signal and perform R-wave detection using a Pan-Tompkins based algorithm^29^. The time differences between consecutive R locations is the inter beat interval (IBI) time series. The IBI time-series is then filtered by removing anomalous values >5 standard deviations that are caused by missed or spurious peak detection. The IHR is calculated as simply the reciprocal of the IBI values. This heart rate time-series is independently normalized for each night by subtracting the mean and dividing by the standard deviation of the night. Finally the time-series is resampled with linear interpolation to a 2 Hz sampling rate and padded with zeros to a constant size corresponding to 10 h or 72,000 elements.

### Algorithm

We used a fully convolutional deep neural network algorithm (Fig. 1). The network can be divided into two distinct parts. The first part employs a CNN which extract local features from the input. The input heart rate time-series (of length 72,000) is broken up into 1200 segments each of length 256 and centered around every 30-s label epoch. All the segments are fed to the convolutional network which is made up of three sequential blocks. Each block is made up of 2 1-D convolutional layers with kernel size 3, dilation rate 1 and leaky RELU activation followed by a maxpool layer of stride 2. Thus after each block the input is downsampled by a factor of 2. The input of each convolutional block is downsampled and added to the output of the block as a residual connection. The output of the final block is flattened and reshaped into a vector of constant length, 128, and then the 1200 segments are concatenated back to an embedding of size 1200 × 128.

The second part of the network employs atrous or dilated convolutional blocks^23,24^ to extract long range features from the input. A dilated block is made of 5 1-D convolutional filters with kernel size 7 each of which is followed by a leaky ReLU activation. The five convolutional layers employ progressively increasing dilation rate of 2, 4, 8, 16, and 32 which are responsible for increasing the network’s field of view. A dropout layer was used after each dilated convolutional block and a residual connection from the input of the block was added to the output of the dropout layer. We use two such dilated convolutional blocks without any pooling layers. Therefore the size of the time axis of the embedding vector remains unchanged at 1200. Finally, a convolutional layer with kernel size 1, dilation rate 1 and 4 filters makes the final output a vector of size 1200 × 4.

### Training

Regularization in the form of L1 weight decay on all convolutional layers and dropout was used to make the model robust to noise in the input. Batch normalization was an optional addition before all convolutional layers, but was not used in the final model configuration. The 1200 × 4 vector is interpreted as 1200 consecutive 4-class probability distributions, one for every 30 s epoch. Loss is calculated as the mean cross entropy loss of the epoch probability distribution vector versus the corresponding label annotated by human experts. The epochs corresponding to the zero padded region of the input are discarded for the loss calculation. The exact values of the hyperparameters such as the number of convolutional blocks, batch size, learning rate, decay rate and dropout rate were chosen based on hyperparameter search. We searched through 1000 configurations of the hyperparameters evaluating the performance of each configuration on the validation set. The best performing model was trained for 1 million steps using a batch size of 2, learning rate of 1e-4, weight decay rate of 0.25, dropout rate of 0.2 with the architecture described above. This configuration was used to train the final model and only this model was evaluated on the test set.

### Performance evaluation

The trained neural network generates four values for every 30-s epoch, corresponding to the probabilities of that epoch belonging to each of the four classes (wake/light/deep/REM). The performance is measured using the 4-class accuracy and Cohen’s kappa metric considering each epoch as an independent prediction and the expert labeled annotations as the reference labels (Fig. 3). Histograms and distributions considering the accuracy of each night as independent statistics are used to determine the bias (or lack thereof) of the model to datasets or patient clinical measures (Fig. 4).

### Statistical analysis

For measures of overall performance (accuracy, kappa and 4-class confusion matrix), the sample sizes are so large that CIs are expected to have a width of <0.5%. Therefore, the CIs are not reported due to the high precision of the values. We report the mean accuracy and mean kappa of the per-night statistics with their respective standard deviations.

The nightly accuracy distributions were stratified by age, gender and presence of apnea and the distributions were compared using pairwise Wilcoxon rank tests without Bonferroni correction. On the SHHS test set, we found no significant difference between the nightly accuracy distribution of male vs female (*p*-value = 0.64) subjects and between healthy vs apneic subjects (*p*-value = 0.68). Increasing age corresponded to a significant decrease in staging accuracy. Comparing the 4 age cohorts, 0 for age <55, 1 for age between 55 and 60, 2 for age between 61 and 70 and 3 for age >70, mean accuracy statistic differed as following—0~1 (*p*-value = 0.3); 0, 1 > 2, 3 (*p*-value < 0.001); 2 > 3 (*p*-value = 0.04).

For clinical measure covariate analyses, we tested the correlation of four sleep metrics—light, deep and REM fraction, and sleep efficiency—against four covariates —age, gender, apnea severity and presence of hypertension. First, each sleep metric was tested separately across the binary covariates (gender and hypertension) using Wilcoxon rank test and across the polychotomous covariates (age and apnea) using Kruskal–Wallis test. We report as significant the measures for which the p-values were below a significance threshold of 0.05 with Bonferroni correction. Plots show the means and standard deviations of each metric-covariate pair where metrics are calculated using both the expert-annotated PSG stages and our algorithm predicted stages (Fig. 5).

**Fig. 6:**
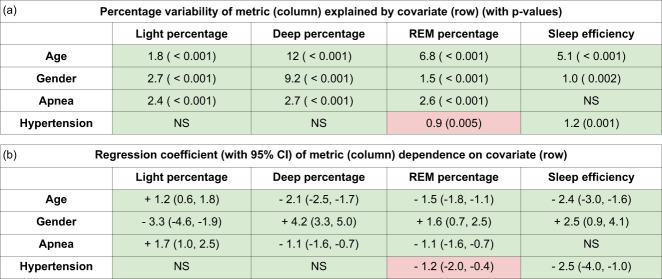
Sleep covariate regression analysis. The tables below show results of regression analysis with the covariates on each row as independent variables and our algorithm predicted sleep metrics on each column as the dependent variable. The metrics are adjusted for age and gender before regressing on apnea, and adjusted for age, gender and apnea before regressing on hypertension. Boxes where the covariate was a significant predictor are filled with values, the rest are filled with “NS”. Boxes colored in green are those where our results qualitatively agree with a previous study^12^ done using on PSG reference stages. Boxes colored in red are those where the previous study^12^ and our results disagree. *N* = 800. **a** Regression coefficients with 95% confidence intervals. **b** Percentage of variance of sleep measure explained by covariate calculated as 100 * R-squared along with *p*-values.

Next, multivariate ordinary least squares linear regression was used to quantify the contribution of various covariates. First, age and gender were entered as independent variables in one model with the sleep metrics as the dependent variable and their significant coefficients were noted. Gender was entered as a binary integer with females as 0 and males as 1. Age was entered as a categorical integer—0 for age < 55, 1 for age between 55 and 60, 2 for age between 61 and 70 and 3 for age > 70. Then the sleep metrics were adjusted for the contribution of age and gender and again used as the dependent variable against the independent covariate apnea severity. Apnea severity was entered as a categorical integer—0 for AHI < 5, 1 for AHI between 5 and 15, 2 for AHI between 15 and 30 and 3 for AHI > 30. Finally, the sleep metrics were adjusted for age, gender and apnea severity and used as dependent variables against the presence of hypertension, which was entered as 0 for absence of hypertension and 1 for presence. For each metric-covariate pair, coefficients from the regression model were reported only if both the *p*-values of the significance test and the regression crossed the significance threshold of 0.05 after Bonferroni correction, the rest were reported as not significant (Fig. 6).

Finally a fifth sleep metric, mean REM bout duration, was regressed against apnea severity. Subjects from three apnea cohorts, mild, moderate, and severe, were considered. Once again, first, age and gender were entered as independent variables with mean REM duration as the dependent variable in a linear regression model. Age was found to have no significant effect, while gender was found to have a strong effect with women having longer REM bout durations with *p*-value = 0.005. Subsequently, mean REM durations were adjusted for the effect of gender and then regressed against apnea severity which was entered as a categorical integer—0 for AHI < 5, 1 for AHI between 5 and 15, and 3 for AHI > 30. This coefficient was found to be significant, with *p*-value = 0.012, implying REM bout durations decrease with increasing apnea severity.

### Inter-beat interval quality

**Fig. 7:**
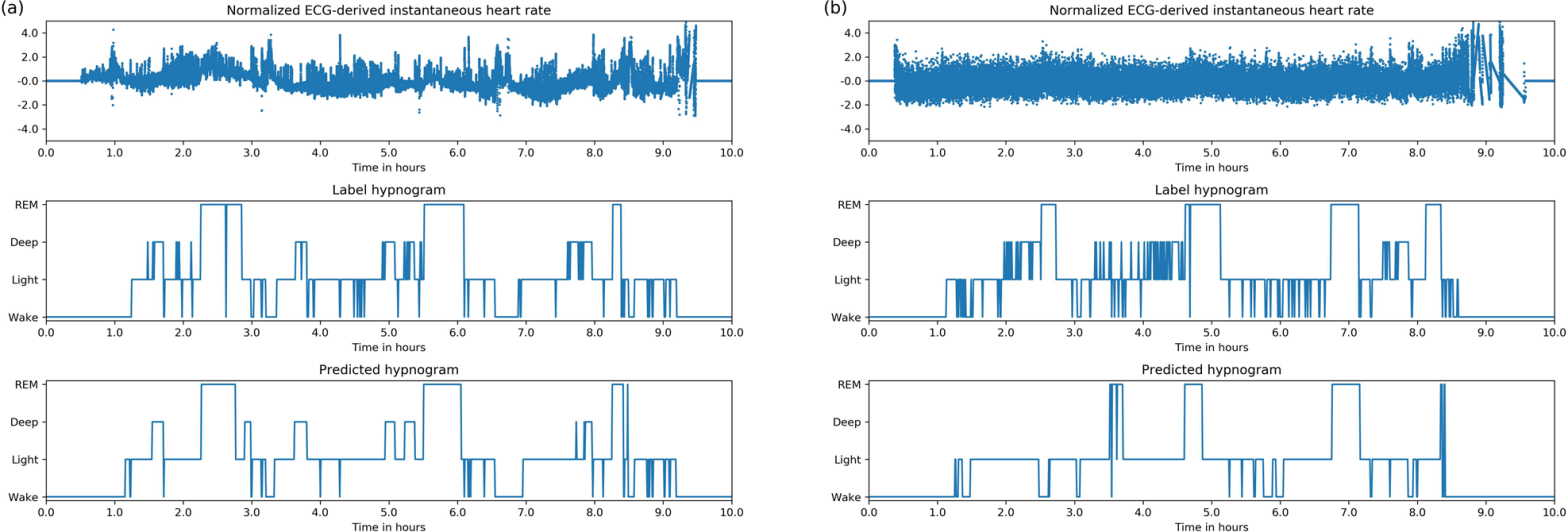
More example hypnograms. **a** Hypnogram of an apneic subject (AHI = 70). Hypnogram shows similar 60–90 min sleep cycles but the cycles often contain significant wake periods. **b** Hypnogram with poor input data quality. Predicted hypnogram gets some of the structure correct but incorrectly classifies many deep and REM epochs.

As seen in the per night accuracy histogram, 10% of the nights are found to have accuracy less than 0.6. We tried to identify the reason why some nights performed worse than others. Upon visual inspection, some of these nights were found to have poor IBI quality (example shown in Fig. 7). However we perform no filtering or exclusion of records based on quality. The reason is twofold: (a) there does not exist a widely accepted criteria of IBI quality that we could use to exclude certain records, and (b) it is possible that poor ECG quality in these studies could be correlated with certain health conditions. Further studies are required to create robust measures of IBI quality and determine if there exists a significant correlation between model performance and IBI quality without confounding it with other comorbidities.

### Demonstration on wrist-worn PPG

**Fig. 8:**
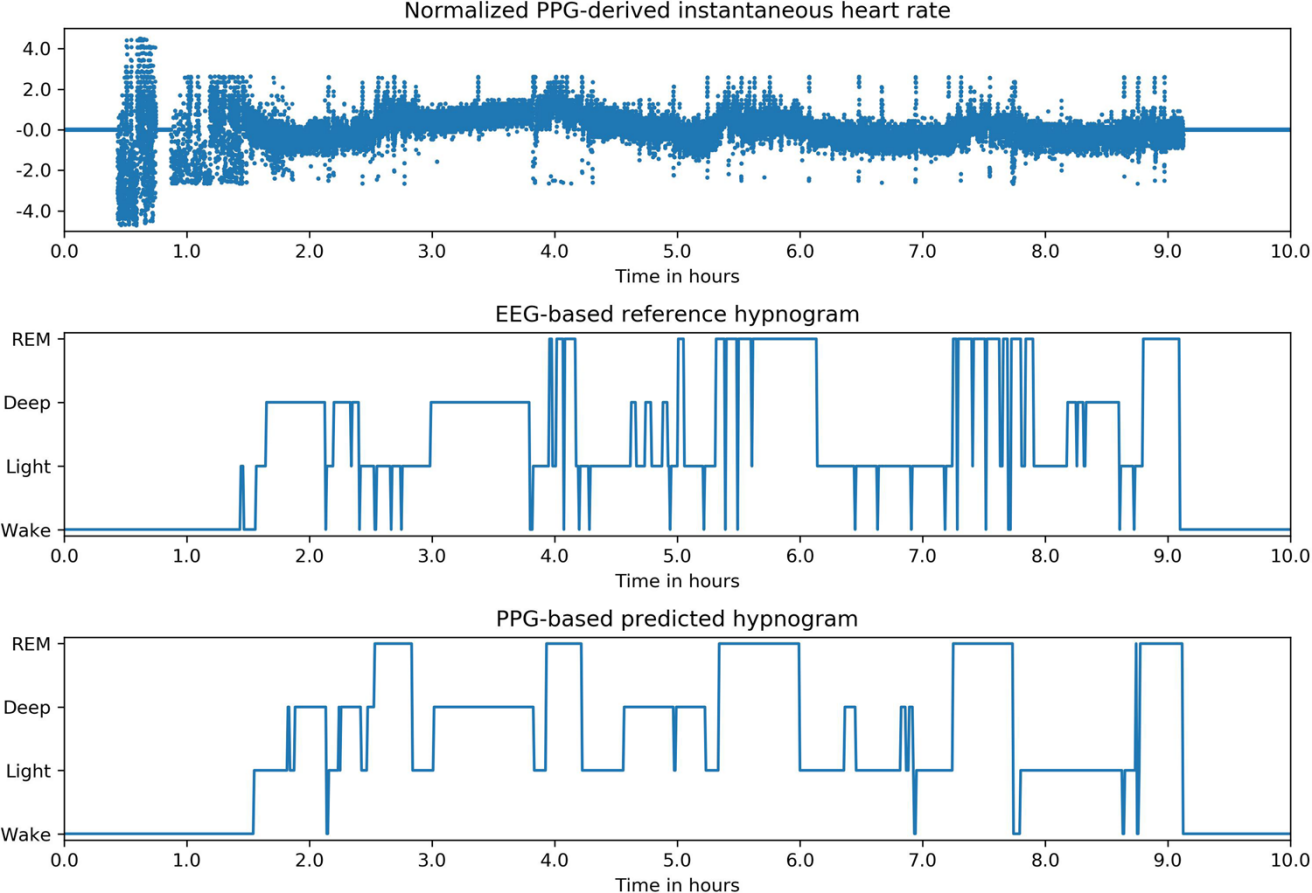
Demonstration on wrist-worn PPG. Example of a hypnogram predicted by our algorithm using heart rate extracted from wrist-worn PPG device compared with the hypnogram predicted by an EEG based automated sleep scoring device.

PPG from wrist-worn devices is one of the most common signals that has found widespread adoption in consumer devices that can yield an accurate IHR signal which has already been shown to be useful for sleep staging^22^. We do not have access to a dataset with PPG data and PSG reference labels for a comprehensive analysis, therefore as a demonstration, we tested our algorithm using a wrist-worn PPG device and ZMachine Insight+, an EEG based automated staging device. We found that the hypnograms predicted by the algorithm using IHR extracted from PPG peaks qualitatively agree with the hypnograms generated by the single-channel EEG-based algorithm developed by ZMachine; an example is shown in Fig. 8. Further studies are required to assess our model’s generalizability to PPG and other signals.

### Reporting summary

Further information on research design is available in the Nature Research Reporting Summary linked to this article.

## Supplementary information

Reporting Summary

## Data Availability

SHHS and MESA have been archived by the National Sleep Research Resource^27,28^ with appropriate de-identification. Permissions and access for these datasets were obtained via the online portal: www.sleepdata.org. The Physionet CinC is archived and available for download at www.physionet.org, released as part of the Computing in Cardiology Challenge 2018^18^.

## Code and software availability

The code used for training the models has a large number of dependencies on internal tooling, infrastructure and hardware, and therefore releasing a binary or working code is not feasible. However, we have detailed all experiments and implementation details in sufficient detail in the “Methods” section to allow independent replication with non-proprietary libraries. The algorithmic components of our work use software that are available in open source repositories: Python 2.7 packages Numpy and Scipy was used for feature extraction and preprocessing. Tensorflow 1.13 was used to build, train and test the model. Python 3.4 packages Numpy, Scipy, Statsmodels, Pandas, Seaborn and Matlplotlib were used to create the plots and statistical analysis.
