# Author Correction: Deep learning for automated sleep staging using instantaneous heart rate

**DOI:** 10.1038/s41746-020-00337-9

**Published:** 2020-10-08

**Authors:** Niranjan Sridhar, Ali Shoeb, Philip Stephens, Alaa Kharbouch, David Ben Shimol, Joshua Burkart, Atiyeh Ghoreyshi, Lance Myers

**Affiliations:** Verily Life Sciences, Mountain View, CA USA

**Keywords:** Machine learning, Biomarkers

Correction to: *npj Digital Medicine* 10.1038/s41746-020-0291-x, published online 20 August 2020

The original version of the published Article contained a typographical error in the spelling of a trademarked name in the Methods. The spelling of ZMachine has been corrected to Zmachine and includes the manufacturer name and location at first mention in the Methods.

The HTML and PDF versions of the Article have been corrected.

