# Supplementary material for: Deep learning for automated sleep staging using instantaneous heart rate
Source: NPJ Digit Med. 2020 Aug 20;3:106. doi: 10.1038/s41746-020-0291-x (PMC7441407; doi:10.1038/s41746-020-0291-x)
Supplement: Supplementary file 1 — Reporting Summary [file 41746_2020_291_MOESM1_ESM.pdf]

nature research

Corresponding author(s): Niranjan Sridhar

Last updated by author(s): Jan 3, 2020

Reporting Summary

Nature Research wishes to improve the reproducibility of the work that we publish. This form provides structure for consistency and transparency in reporting. For further information on Nature Research policies, see Authors & Referees and the Editorial Policy Checklist.

Statistics

For all statistical analyses, confirm that the following items are present in the figure legend, table legend, main text, or Methods section.

n/a	Confirmed	
☐	☒	The exact sample size (n) for each experimental group/condition, given as a discrete number and unit of measurement
☐	☒	A statement on whether measurements were taken from distinct samples or whether the same sample was measured repeatedly
☐	☒	The statistical test(s) used AND whether they are one- or two-sided<br>*Only common tests should be described solely by name; describe more complex techniques in the Methods section.*
☐	☒	A description of all covariates tested
☐	☒	A description of any assumptions or corrections, such as tests of normality and adjustment for multiple comparisons
☐	☒	A full description of the statistical parameters including central tendency (e.g. means) or other basic estimates (e.g. regression coefficient) AND variation (e.g. standard deviation) or associated estimates of uncertainty (e.g. confidence intervals)
☐	☒	For null hypothesis testing, the test statistic (e.g. F, t, r) with confidence intervals, effect sizes, degrees of freedom and P value noted<br>*Give P values as exact values whenever suitable.*
☒	☐	For Bayesian analysis, information on the choice of priors and Markov chain Monte Carlo settings
☒	☐	For hierarchical and complex designs, identification of the appropriate level for tests and full reporting of outcomes
☒	☐	Estimates of effect sizes (e.g. Cohen's d, Pearson's r), indicating how they were calculated

Our web collection on statistics for biologists contains articles on many of the points above.

Software and code

Policy information about availability of computer code

Data collection	Python 2.7 packages Numpy and Scipy were used for data ingestion, preprocessing and feature extraction.
Data analysis	Tensorflow 1.14 was used to build, train and test the model. Python 3.4 packages Numpy, Scipy, Statsmodels, Pandas, Seaborn and Matplotlib were used to create plots and analyze the results.

For manuscripts utilizing custom algorithms or software that are central to the research but not yet described in published literature, software must be made available to editors/reviewers. We strongly encourage code deposition in a community repository (e.g. GitHub). See the Nature Research guidelines for submitting code & software for further information.

Data

Policy information about availability of data

All manuscripts must include a data availability statement. This statement should provide the following information, where applicable:

- Accession codes, unique identifiers, or web links for publicly available datasets
- A list of figures that have associated raw data
- A description of any restrictions on data availability

The Sleep Heart Health Study and Multi-Ethnic Study of Atherosclerosis data were downloaded from the National Sleep Research Resource website (www.sleepdata.org). The data is available by obtaining permission through their web portal. The Computing in Cardiology dataset is available for download from Phyionet (www.physionet.org). All datasets have already been de-identified by the source.

Field-specific reporting

Please select the one below that is the best fit for your research. If you are not sure, read the appropriate sections before making your selection.

☒ Life sciences ☐ Behavioural & social sciences ☐ Ecological, evolutionary & environmental sciences

For a reference copy of the document with all sections, see nature.com/documents/nr-reporting-summary-flat.pdf

Life sciences study design

All studies must disclose on these points even when the disclosure is negative.

Sample size	Sample sizes was pre-determined for us by our choice of public datasets. Our SHHS test set size was decided by a random choice which allots approximately 80% of the dataset for training, 10% for validation and 10% for test set. The exact number of nights(subjects) in the test set is reported. The Physionet CinC dataset is used in its entirely as a test set and thus was pre-determined. Therefore while we do not pre-specify a size for our test set, we report the largest test dataset used by previous studies on the same dataset.
Data exclusions	No data was intentionally excluded from the dataset. We determine however that less than 0.5% of the dataset might have been excluded accidentally due to data corruption during download and transfer of data.
Replication	The model accuracy achieved on the training datasets was verified by testing on large, completely new dataset that was collected in a different source, population and time period. The results on the new data matched very closely with the results on the datasets used to build the model, giving us confidence in the reproducibility of our results.
Randomization	All training dataset (SHHS and MESA) records were obscured by a 128bit hash based on their de-identified serial number and then randomly split into training, test and validation set based on the value of the hash. This was only done once and the datasets were never mixed. The training dataset was randomly shuffled during the training process. The final external test set (CinC) was never split and the entire dataset was used for testing with no randomization. No covariates were controlled in this study.
Blinding	Blinding was not relevant to this study as we did not modify the study data based on the groups of data, all data regardless of source or grouping was processed in the same manner.

Reporting for specific materials, systems and methods

We require information from authors about some types of materials, experimental systems and methods used in many studies. Here, indicate whether each material, system or method listed is relevant to your study. If you are not sure if a list item applies to your research, read the appropriate section before selecting a response.

Materials & experimental systems

n/a	Involved in the study	
☒	☐	Antibodies
☒	☐	Eukaryotic cell lines
☒	☐	Palaeontology
☒	☐	Animals and other organisms
☒	☐	Human research participants
☒	☐	Clinical data

Methods

n/a	Involved in the study	
☒	☐	ChIP-seq
☒	☐	Flow cytometry
☒	☐	MRI-based neuroimaging
